# A study to determine the prevalence and factors associated with hypertension among employees working at a call centre Nairobi Kenya

**DOI:** 10.11604/pamj.2017.27.178.13073

**Published:** 2017-07-05

**Authors:** Mwagi Joseph Onyango, Iyeri Kombe, Daniel Sagwe Nyamongo, Moses Mwangi

**Affiliations:** 1 Jomo Kenyatta University of Agriculture and Technology, College of Health Sciences, Nairobi, Kenya; 2 Kenya Medical Research Institute, Nairobi, Kenya

**Keywords:** Hypertension, body mass index, health risk factors

## Abstract

**Introduction:**

Hypertension often referred to as Non Communicable Diseases (NCDs). Causes of hypertension are classified into modifiable and non-modifiable factors. The objective of the study was to determine the prevalence and other associated factors leading to the onset of hypertension among employees working at the call center.

**Methods:**

This was a descriptive cross sectional study design. Data collection was done in two parts; part one comprised of clinical health assessments; weight and height to aid determine Body Mass Index and blood pressure measurement. Part two was by self-administered questionnaires to participants to aid identify behavioral risk factors and further elicit lifestyle practices. Data was collected from a sample population of 370 respondents. Descriptive statistical analysis was applied in univariate analysis. Further analysis included bivariate and multiple regression analysis; Odds Ratio with 95% confidence interval was used to determine the strength of association.

**Results:**

The proportion of hypertension was significantly higher among overweight respondents (32.7%) (OR= 11.55; 95% CI= 4.44-30.07; P < 0.001) and obese respondents (60.2%) (OR= 36.02; 95% CI= 13.43-96.60; P < 0.001) compared to those respondents who were within normal range of weight (4.0%). Nine (9) factors that were associated with hypertension at bivariate analysis (P < 0.05) were all subjected to a multiple regression analysis or reduced model where four factors remained in the final analysis. Respondents who were classified as overweight had 10.6 times likelihood developing hypertension compared to those respondents with normal weight (AOR= 10.61; 95%CI= 3.98-28.32; P < 0.001). Likewise, obese respondents were 43.6 fold more likely to develop hypertension compared to those respondents within normal range of weight [OR=43.68; 95%CI=15.24-125.16; P<0.001]. Respondents not trying to reduce fat in their diet were highly predisposed having hypertension at (AOR=2.44; 95% CI=1.20-4.96; P= 0.014) than respondents who always tried to reduce fat in their diet. Respondents who sometimes engage on more physical exercises were 2.2 times likely to develop hypertension (AOR=2.22; 95%CI= 1.20-4.10; P= 0.011) compared to those who always engaged in more physical exercises. Respondents with parenting issues were about twice as likely to have hypertension (AOR= 2.15; 95% CI: 1.23-3.74; P= 0.007) than parents who did not have parenting issues.

**Conclusion:**

This study depicts rising cases of hypertension and an alarming rate of pre-hypertension among the working population. This vary based on the age, obesity, parental responsibility, unhealthy diet and lack of or reduced physical activity. These call for strategic interventions and greater emphasis on health promotion programs at the workplace alongside staff empowerment towards health seeking behaviors.

## Introduction

Globally all nations have committed to reduce premature mortality arising from Non Communicable Diseases (NCDs) by 25% by the year 2030 [1]. This declaration was reached after World Health Organization global report indicated that out of 57 million deaths; 36 million (63%) were due to non-communicable diseases majorly; mainly hypertension, stroke and heart attack, diabetes, cancer and chronic respiratory disease. Sadly, these are projected to reach 80% of major cause of death by the year 2030 [1]. A large percentage of non-communicable diseases are preventable through the reduction of the four main modifiable behavioural risk factors; Unhealthy diet, physical inactivity, harmful use of tobacco and excessive alcohol consumption and reducing obesity. Among the adopted strategies are the “Alameda seven” [2] are linked with better health. These are eating healthy breakfast, maintaining proper weight, not snacking in between meals, never smoking cigarettes, regular physical activity, moderate or no use of alcohol and getting adequate and regular 7-8 hours of sleep.

**Global perspective**: According to World Health Organization [3] approximately 40 percent of adults aged 25 and above had been diagnosed with hypertension. The number of people with hypertension rose from 600 million in 1980 to one billion in 2008 [3]. The prevalence of Hypertension is highest in the African region at 46 percent [1]. Further the numbers of people with undiagnosed, untreated and uncontrolled hypertension are also high in lower and middle income countries compared to high income countries [1]. Hypertension was mainly associated with well to do and affluent regions in the world. However, studies indicate that hypertension is increasingly becoming an issue in low and middle income countries due to scarce health resources which are stretched by infectious diseases burden [4]. Hypertension, otherwise known as “high or raised” blood pressure is a global public health issue. Hypertension is the leading cause of cardiovascular diseases (CVDs) worldwide [4]. It contributes to the complications associated with diseases of the heart; stroke, kidney failure and premature mortality [3]. Most people with hypertension rarely show symptoms at early stages and consequently most people go undiagnosed. Globally hypertension is responsible for at least 45 percent of deaths due to heart disease and 51 percent deaths due to stroke. A report [5] shows that rising medical claim trend is a global trend and a challenge to affordable universal healthcare provision. Rand corporation [6] shows that rising medical claims is on the rise across the globe and most insurers see no end in sight. Rand corporate research contends that no challenge in healthcare is more important than reducing healthcare spending especially those associated with preventable risk factors. According to Kaiser survey [7], the cost of health insurance has leapfrogged to the second most largest expense beyond payroll for most employers and that the average premium for a family insurance coverage increased to 131% since 1999 [8]. This healthcare quandary is attributed to chronic diseases such as hypertension, heart disease, cancer, stroke, and diabetes. However, healthcare costs will always be on the rise until such a time when employers will invest heavily in health promotion such as prevention, early diagnosis or rehabilitation of persons with chronic diseases such as hypertension [9].

**Causes of hypertension**: Risk factors associated with onset of hypertension are classified into four groups [1] while CDC summarises the risk factors into modifiable and non-modifiable [10]. According to WHO [1] the four major risk factors are behavioural risk factors, metabolic factors, social determinants and cardiovascular diseases. The behavioural risk factors [1] associated with the development of hypertension includes; unhealthy diet, tobacco use, physical inactivity and harmful use of alcohol. The metabolic risk factors such as High blood pressure, obesity, diabetes and raised blood lipids. Social determinants include; globalization, urbanization, ageing, education and housing. Lastly, cardiovascular diseases that are directly related to hypertension are heart attacks, strokes, and heart failure and kidney diseases. While most hypertensive people have no symptoms at all [3], it is important for everybody to know their blood pressure readings and watch out for symptoms associated with high blood pressure such as; headache, shortness of breath, dizziness, chest pains, palpitations of the heart and nose bleeding [11]. World Health Organization [3] has defined the need to develop a national database for hypertension screening. This will aid in developing target interventions to reduce NCD's associated with the rise in hypertension Methods. The study had three-fold objectives; to determine prevalence of hypertension among employees working at the call centre, to establish behavioural health risk factors leading to hypertension among the employees and to identify healthy lifestyle practices among employees.

## Methods

**Study design**: This was a descriptive cross sectional study. Simple random sampling was used to identify the study participants.

**Study area**: The study was carried out at a communication company situated in Nairobi county in Kenya. The communication company is situated along the Nairobi-Mombasa highway, i.e. on your way to Mombasa approximately 20km from the city (Nairobi) centre. The company was chosen following the management request to their medical administrator to establish the causes leading to the increased cases of hypertension among staff attending the in-house medical facility. The company has a workforce of 1600 employees out of which 370 participated in the study.

**Study population**: The study population was 1600 staff working in the company. A total sample population of 400 staff was sampled, however only 370 respondents completed the process.

### Eligibility criteria

**Inclusion Criteria**: Permanent staff employed at the communication company, who also gave written consent to participate in the study voluntarily.

**Exclusion criteria**: Permanent staff who did not sign consent to participate in the study; temporary staff; contract staff; interns; any staff below 18 years of age.

**Sample size**: The Fisher et al 1998, formula was used to calculate the minimum required sample size as follows: Where n= Minimum required sample size α= Level of significance (0.05) Z_α/2_= Standard normal deviate at 95% CI (1.96) P = Assumed prevalence of Hypertension among employees (50%). d= Absolute precision (Error margin),(0.05) n =385 385+15=400 (An additional 10% of sample was therefore 400 participants were recruited in the study to take care of non-respondents and refusals).

**Sampling procedure**: The sampling frame consisted of a list of 1600 staff comprising a list of all the staff in the organization. From the staff list, a sample size of 400 was selected. A computer generated random numbers was used to select the 400 participants by use of simple random sampling method.

### Recruitment and consenting

**Recruitment of participants into the study**: This was done on voluntary basis. Information about the study and its benefits was explained to individual participants. Confidentiality was upheld by use of codes for anonymity purposes. Opt in-opt out approach was used during the study period.

**Pre-testing**: This was done at the company's in-house clinic. This was to assist in aligning the questionnaire with the intended purpose and further determine the response time taken for each participant.

**Variables dependent:** Blood pressure measurement.

**Independent variables**: Socio-Demographic factors: age; gender; marital status; level of education Behavioral risk factors; diet; exercise; rest and sleep; alcohol consumption and tobacco smoking physical parameters (weight/body mass index).

**Data collection process**: The study was conducted between August 2016 and December 2016. This was done in two parts;

**Clinical assessments for baseline tests**: Weight and height were taken to aid determine the Body Mass Index and blood pressure measurements.

**Data collection**: A structured self-administered questionnaire was used to collect data on behavioral risk factors. The questionnaire was developed from the emerging themes in the literature review according to the World Health Organization's (STEPS) format.

### Data management and analysis plan

**Data management**: Data was done by use of version 20.00 (SPSS) format, upon data entry, data cleaning and validation before being exported to into the statistical software.

**Data analysis plan:** Data analysis was done in two steps; STEP 1(behavioural risk factors); stratification of specific behavioural health risk predisposing factor by (demography) individual characteristics (age, gender, education, marital status,) was done. Further analyses included prevalence estimates for specific behavioural health risk predisposing factors (tobacco use, alcohol consumption, physical inactivity and unhealthy diet), with their 95% confidence interval. Chi-square or Fisher exact statistics was used to test associations between individual characteristics and specific behavioural health risk predisposing factors at bivariate analysis. Strength of association was determined based on odds ratio at 95% confidence interval. All individual characteristics identified to be significantly associated with specific behavioural health risk predisposing factor at p<0.05 during bivariate analysis were considered for multivariate analysis. Calculation of Adjusted Odds Ratio (AOR) based on 95% confidence interval was done using binary logistic regression, for significant individual characteristics (age, gender, education, marital status). Step 2 (analysis of physical parameters); analyses include prevalence estimates for specific physically measured health risk predisposing factor (overweight/obese and high blood Pressure). Their 95% confidence interval were determined. Further stratification of the specific physically measured health risk predisposing factor were done by individual characteristics (age, gender, education, marital status) and specific behavioural health risk predisposing factor determined at STEP 1. The specific physically measured health risk predisposing factor (overweight/obese and high blood pressure) in relation to individual characteristics and specific behavioural health risk predisposing factor determined at STEP 1 were tested using chi-square or Fisher exact statistics. Odds ratio at 95% confidence interval was used to determine the strength of association. All individual characteristics and specific behavioural health risk pre-disposing factor determined at STEP 1, identified to be significantly associated with specific physically measured health risk predisposing factor (overweight/obese and high blood pressure) at p<0.05 during bivariate analysis were considered for multivariate analysis. Similarly, Adjusted Odds Ratio (AOR) at 95% confidence interval was calculated using binary logistic regression, where significant individual characteristics (age, gender, education, marital status) and specific behavioural health risk pre-disposing factors determined at STEP 1 were included simultaneously in the model, in order to assess the determinants of physically measured specific health risk predisposing factor (overweight/obese and high blood pressure). A p-value <0.05 was considered as statistically significant. Step 3 (health seeking behaviours) This analysed the responses from participants on the health seeking behaviours with regard to selected activities for implementation at the organization. Summary of responses were compared to the demographical representation (age, gender, education, marital status) as in step.

**Ethical consideration**: Ethical approval for the study was obtained from the Kenyatta National Hospital University of Nairobi Ethics and Research Committee. This was granted and signed by the appointed medical administrator (on behalf of the company) upon whose approval the researcher was granted the permission to carry out the study. Eligible study participants signed consent prior to participation in the study. Confidentiality of participants and related information was upheld. This was highly protected by use of codes on the questionnaires to reduce chances of disclosure. Anthropometric measurements used blood pressure machine, weight and height meter. Self-administered questionnaire was given to participants by the research assistants Training of research assistants was facilitated prior commencement of the study. Anthropometric assessments instruments were calibrated by an approved agency. Pre-testing of the questionnaire was facilitated to align it with the intended purpose.

## Results

A total of 370 employees working at the communication company in Nairobi were consented to participate in the study (Table 1). Male were 34.9% while female were 65.1%. married 59.5%, single 36.2%, while divorced, single, separated and widowed were 4.4% cumulative. Level of education; Diploma graduate 39.7%, undergraduate 48.4% while post graduate degree were 11.9%. Body Mass index findings; Normal 33.5%, overweight 41.4%, while obese 25.1%, blood pressure findings; the prevalence of hypertension in this study were as follows; normal blood pressure 17.0%; pre-hypertension 53.0%; hypertension stage 1-13.5%; hypertension stage 2-16.2%. The mean systolic blood pressure was 125.8 with a standard deviation of 14.5 and diastolic blood pressure was 83.9 with a standard deviation of 11.0 (Table 1). There was a significant association between increased body mass index and hypertension. Respondents aged 40 to 49 years were significantly more likely to develop hypertension (50.0%) (OR= 2.72; 95%CI= 1.13-6.55; P= 0.025) compared to those respondents aged 18 to 29 years (26.9%). The proportion of hypertension was significantly higher among overweight respondents (32.7%) (OR= 11.55; 95% CI= 4.44-30.07; P < 0.001) and obese respondents (60.2%) (OR= 36.02; 95%CI= 13.43-96.60; P < 0.001) compared to those respondents who were within normal range of weight (4.0%) (Table 2). There was significantly increased proportion of hypertension among respondent with family history of high blood pressure (36.3%) (OR= 1.64; 95%CI= 1.03-2.63; P= 0.038) than to those without family history of high blood pressure (25.8%) (Table 2). Respondents who did not eat at least five servings of fruits and vegetables each day had significantly increased proportion of hypertension (37.3%) [OR=1.75; 95%CI=1.03-2.96; P=0.037] compared to respondents who indicated otherwise (25.4%). Similarly, there was increased proportion of hypertension among respondents who were not trying to reduce consuming fat in the diet (50.8%) (OR= 2.71; 95%CI= 1.52-4.83; P= 0.001) than to those who were always trying to reduce fat (27.6%) (Table 2). Respondents who engaged sometimes on more physical exercises engagement had significantly more proportion of hypertension (41.8%) (OR=2.20; 95%CI=1.34-3.61; P=0.002) than those who always engaged in more physical exercises (24.6%) (Table 2).

**Table 1:** Hypertension in relation to socio-demographic characteristics

Variables	Hypertensive, (N=111)		No hypertension, (N=259)		OR ^ψ^	95% CI ^φ^		p value*
	n	%	n	%		Lower	Upper	
**Age**								
18-29 Years	18	26.9%	49	73.1%	Reference			
30-39 Years	77	28.4%	194	71.6%	1.08	0.59	1.97	0.801
40-49 Years	16	50.0%	16	50.0%	2.72	1.13	6.55	0.025
**Gender**								
Male	45	34.9%	84	65.1%	1.42	0.90	2.25	0.134
Female	66	27.4%	175	72.6%	Reference			
**Marital Status**								
Single	43	32.1%	91	67.9%	Reference			
Married	63	28.6%	157	71.4%	0.85	0.53	1.35	0.492
Others (Separate, divorce, widowed)	5	31.2%	11	68.8%	0.96	0.32	2.94	0.946
**Level of education**								
Diploma Graduate	47	32.0%	100	68.0%	Reference			
Undergraduate (Bachelor's Levels)	48	26.8%	131	73.2%	0.78	0.48	1.26	0.308
Postgraduate (Masters' Level)	16	36.4%	28	63.6%	1.22	0.60	2.46	0.587
**Body mass index classification**								
Normal weight (18.5-24.9)	5	4.0%	119	96.0%	Reference			
Overweight (25 - 29.9)	50	32.7%	103	67.3%	11.55	4.44	30.07	<0.001
Obese (=>30)	56	60.2%	37	39.8%	36.02	13.43	96.60	<0.001

**Table 2:** Bivariate analysis of association between selected socio-demographic characteristics and hypertension

Variables	AOR	95% CI		p value*
		Lower	Upper	
**Full model**				
**Age**				
18-29 Years	Reference			
30-39 Years	0.77	0.36	1.66	0.508
40-49 Years	2.67	0.86	8.35	0.091
**Body mass index classification**				
Normal weight (18.5-24.9)	Reference			
Overweight (25 - 29.9)	10.75	3.87	29.90	<0.001
Obese (=>30)	48.45	15.81	148.47	<0.001
**Family history of increased Blood pressure (high blood Pressure)**				
Yes	1.39	0.74	2.60	0.301
Don’t Know	1.24	0.21	7.41	0.815
No	Reference			
**Eat at least five servings of fruits and vegetables each day**				
No	1.64	0.78	3.46	0.193
Sometimes	1.17	0.51	2.67	0.717
Yes	Reference			
**I often try to reduce fat in my diet**				
No	2.01	0.94	4.30	0.074
Sometimes	0.19	0.07	0.48	<0.001
Yes	Reference			
**Start or engage in more physical exercises/activities**				
No	1.21	0.50	2.95	0.679
Sometimes	1.82	0.91	3.64	0.088
Yes	Reference			
Undertake a heart function test				
Yes	Reference			
Sometimes	2.75	0.91	8.30	0.074
No	1.78	0.82	3.87	0.148
**Network with focused groups on well-being e.g. building healthy families**				
Yes	Reference			
Sometimes	1.61	0.65	3.99	0.305
No	0.89	0.44	1.78	0.732
**Parenting issues**				
Yes	1.63	0.88	3.00	0.119
No	Reference			

Bivariate analysis of association between substance use and hypertension showed that there was no significant association observed between the variables (Table 2). There was increased proportion of hypertension among respondents who indicated undertaking heart function test sometimes (40.0%) (OR= 2.58; 95%CI: 1.15-5.80; P=0.022) and not at all (30.0% OR= 1.83; 95%CI: 1.01-3.32; P= 0.048) compared to those who indicated undertaking the test always (20.0%). Respondents who rated network with focused groups on well-being as sometimes had significantly more proportion of hypertension (46.4%) [OR=2.34; 95%CI: 1.21-4.52; P= 0.012) than those who rated always (27.0%) (Table 2). Respondents with parenting issues had significantly increased proportion of hypertension (39.3%) (OR= 2.05; 95%CI: 1.31-3.22; P= 0.002) compared to those who indicated otherwise (24.0%) (Table 2). Nine (9) factors which associated with hypertension at P<0.05 during bivariate analysis were subjected all together in a multiple regression analysis upon fitting these factors using binary logistic regression and by specifying “backward LR” method with removal at P<0.05, four (4) factors remained in the final analysis or reduced model (Table 3). Multiple regression analysis identified factors independently associated with hypertension among the respondents (Table 3). Respondents who were classified as overweight had 10.6 times likelihood of developing hypertension as compared to those respondents with normal weight (AOR= 10.61; 95%CI= 3.98-28.32; P < 0.001). Likewise, obese respondents were 43.6 fold more likely to develop hypertension compared to those respondents within normal range of weight (OR= 43.68; 95%CI= 15.24-125.16; P < 0.001) (Table 3). Respondents not trying to reduce fat in their diet were 2.4 times more likely to have hypertension (AOR= 2.44; 95%CI= 1.20-4.96; P= 0.014) than to those always tried to reduce fat in their diet (Table 3). Respondents who sometimes engage on more physical exercises had 2.2 chance of developing hypertension (AOR= 2.22; 95%CI= 1.20-4.10; P= 0.011) compared to those who always engaged in more physical exercises (Table 3). Respondents with parenting issues were about 2 times more like to have hypertension (AOR= 2.15; 95%CI: 1.23-3.74; P= 0.007) compared to those who did not have parenting issues (Table 3).

**Table 3:** Multivariable analysis of factors associated with hypertension

Reduced model				
**Body mass index classification**				
Normal weight (18.5-24.9)	Reference			
Overweight (25-29.9)	10.61	3.98	28.32	< 0.001
Obese (= or >30)	43.68	15.24	5.16	< 0.001
**I often try to reduce fat in my diet**				
No	2.44	1.20	4.96	0.014
Sometimes	0.58	0.43	1.61	0.091
Yes	Reference			
**Start or engage in more physical exercises/activities**				
No	1.15	0.51	2.58	0.741
Sometimes	2.22	1.20	4.10	0.011
Yes	Reference			
**Parenting issues**				
Yes	2.15	1.23	3.74	0.007
No	Reference			

AOR = Adjusted odds ratio; CI = Confidence interval

## Discussion

This study indicates a rising trend of hypertension (30%). Notably there was a high prevalence on pre-hypertensive respondents (53%). According to Bonita et al [12], WHO steps survey conducted between 2003-2009 in 20 African countries reported a prevalence rate of between 19.3% and 39.6% between countries. This was being considered a time bomb in waiting. It is recognized that persons with pre-hypertension are at a much higher risk of progressing to hypertension and becoming prone to hypertension associated CVDs [13] estimates that 30% conversion rate from pre-hypertension state to hypertension every four years [3]. classifies causes of hypertension into four major categories; behavioral risk factors, metabolic factors, social determinants and cardiovascular diseases, while [7] summarizes the risk factors into modifiable and non-modifiable factors. According to Ruseski [14] physically inactive people are more likely to be obese which is a risk factor for NCDs including high blood pressure, WHO [3] reports that approximately 2.3 million deaths occur each year from harmful use of alcohol. Smoking is estimated to cause about 71% of lung cancer [3] while tobacco use increases the risk of complications of hypertension. From this study finding, the associated factors leading to the high prevalence rate of hypertension among employees were: age in years, body mass index, and family history associated with hypertension, physical inactivity and unhealthy diet. Other health seeking factors included; failure to seeking health services e.g. heart function tests, respondents linked with inaccessibility to a network of focused group. Respondents with parenting responsibility were at higher risk of developing hypertension. The study finding indicated that adults within the ages 40-49 years old were more at risk of developing hypertension as compared to others. WHO steps survey further reported that hypertension cases were more pronounced among adult population of 18 years and above. According to World Health study [15] hypertension is more prevalent in urban population than in the rural population. The same study found that advancement in age is a contributing factor to onset of hypertension. Therefore demographic shift [4] is expected to contribute to the high prevalence of high blood pressure among population in the years to come. From the study, overweight and obesity (60.2%) was attributed to the onset of hypertension compared to those of normal weight. Obesity [3] refers to the accumulation of excess body fat in the body and its known to adversely affect the health of an individual. The degree of body weight is usually expressed as BMI; the ratio of weight in kilograms to the square of height in meters. Obesity increases the risk of hypertension and has also been associated with coronary artery disease and some cancers [14]. Obesity is associated with reduced life expectancy [14]. WHO Steps survey findings [15] shows that overweight and obesity is highly prevalent in African countries. Notably, unhealthy diet had a significant increase in proportion of hypertension among respondents (37.3%) who did not eat at least five servings of fruits and vegetables compared to those who indicated otherwise (25.4%). Similarly increased proportion of hypertension was noted among respondents who were not trying to reduce consuming fat in the diet (50.8%) than those who were trying to reduce fat (27.6%). Fruits and vegetable are considered a healthy dietary habit whose benefits are associated with reduced risk of hypertension and other related CVDs. Unfortunately fruits and vegetable consumption is affected by economic, cultural and the potential agricultural productivity [16]. The WHO steps surveys advocates for sufficient fruits and vegetable intake and defined it as five or more servings per one typical day [12].

The association between physical exercise and hypertension [12] revealed that respondents who engaged sometimes in more physical exercise had significantly more proportion of hypertension (41.8%) compared to those who were engaged always in more physical exercise (24.6%). Evidence [17] shows that physical activity has positive on reducing hypertension. WHO steps findings prescribes physical activity as being moderately or vigorously engaged for more than 150 minutes per week. Adequate physical activity has been shown to have many health promoting effects and has a direct, independent role in reducing hypertension [12]. From this study, there was no significant association between substance use and hypertension. This could be attributable to the small sample size; it was difficult to disentangle this variable. However, studies [18] indicate that tobacco smoking is associated with the risk of developing hypertension [12]. Further, tobacco smoking predisposes to cardiovascular diseases such as stroke, thrombosis, and heart attack [17]. WHO steps survey [12] identified other socio-factors that significantly increase the risk of hypertension such as high level (excessive) consumption of alcohol; Respondents with a family history of hypertension (36.3%) had a significantly increased proportion of hypertension compared to those without family history of high blood pressure (25.8%). World Heart Federation [19] contends that history of presence of cardiovascular disease in a family increases an individual's risk to developing hypertension. Further, respondents who rated network with focused groups on well-being as sometimes had significantly more proportion of hypertension (47.4%) than those who rated always (27.0%). Social networks supports [20] provide and keep communication channels open, social networks may also shape disease diagnosis and management. Stronger network ties-as measured by frequency of interaction and emotional closeness-buffer physiological responses to stress and reduce feelings of loneliness which can give rise to physiological dysregulation such as inflammation and elevated blood pressure On life challenges, an analysis of relationships between life challenges and hypertension revealed that respondents with parenting issues had slightly increased proportion of hypertension (39.3%) as compared to those who indicated otherwise (24.0%). A correlational study on mental health [21] indicated reported a significant relationship among parental stress and health as attributable to childcare characteristics and social support. The study further that health is adversely affected as a result of parental stress associated with child care and support The substantial variation in the prevalence of hypertension remained in the multi-variable analysis. This variation seems to have at least two important consequences; the first is that it would be inconsiderate to make estimate based on small sample size, it was difficult to disentangle the various determinants of hypertension among the study group, these include level of education, marital, level of income, unmeasured stress levels among others. Thus the finding can only be generalized to a similar population group. Secondly, that there must be other risk factors of fundamental importance for hypertension other than those analyzed here. Therefore, further investigative studies need to be carried out to define proactive care which is viewed as both logical and necessary alternatives to traditional healthcare approaches [9]. Until promotive health approaches are embraced and implemented at the workplace, hypertension will continue to be cost driver [22] in many African countries.

## Conclusion

In conclusion, this study depicts a greater emphasis on integrated health management program that incorporates preventive health and advocacy programs towards modifiable behavioral risk factors such as diet, exercise, and obesity. Emphasis on staff empowerment towards health seeking behaviors is paramount to the success of alleviating rising cases of hypertension. Other strategic approaches towards preventing the rising cases of hypertension include; policy formulation and emphasis on healthy diet, physical activity reduction of alcohol consumption and avoidance of tobacco consumption. Other strategic approaches are; improved health service delivery, especially at the in-house clinic as the initial entry point for those seeking health services. The need to integrate preventive health services into the medical insurance plan cannot be abated. Improved quality of care and management for the management of chronic NCDs and overall prevention of complications arising as a result of hypertension.

### What is known about this topic

- Hypertension is a silent killer disease that can be diagnosed upon physical measurement of blood pressure;
- Hypertension is a non-communicable disease which is preventable through behavioral modification practices;
- Hypertension is slowly becoming an issue in low and middle income countries whose prevalence is highest in Africa region.

### What this study adds

- The study will elicit factors associated with hypertension among employees working at a call centre;
- The study will highlight on the relation of hypertension to socio-demographic characteristics;
- The study will establish association between selected socio demographic characteristics and hypertension.

## Competing interests

The author's declare no competing interest.
